# Would the Use of Artificial Intelligence in COVID-19 Patient Management Add Value to the Healthcare System?

**DOI:** 10.3389/fmed.2021.619202

**Published:** 2021-01-27

**Authors:** Manuel Cossio, Ramiro E. Gilardino

**Affiliations:** ^1^Artificial Intelligence Master's Program, Faculty of Informatics, Catalonian Polytechnic University, Barcelona, Spain; ^2^HE-Xperts Consulting LLC, Miami, FL, United States

**Keywords:** SARS-CoV-2, COVID-19, artificial intelligence, machine learning, health systems, value based healthcare

## Introduction

Since late 2019, when emerged from Wuhan, the severe acute respiratory syndrome coronavirus 2 (SARS-CoV-2) encircled the globe originating the coronavirus disease 2019 (COVID-19) pandemic (1). Up to December 5, it infected 65 million people, and it caused 1.5 million deaths (2). While 80% of patients present mild symptoms, 20% may experience more severe symptoms that require strict follow-up and hospitalization. Further, about 28–30% of these hospitalized patients will be admitted to the intensive care unit (ICU) (3).

COVID-19 burdened healthcare systems worldwide and changed the paradigm of providing patient care to maximize efficiency and prevent staff members' transmission, which might decrease the workforce to manage the surge.

The use of Artificial Intelligence (AI) gained attention during the COVID pandemic; there are many examples, including the use of mathematical modeling to understand the disease epidemiology, tracking cases, or even supporting decision-makers in pandemic planning (4–6). It's worth mentioning the case of The Center for Systems Science and Engineering at Johns Hopkins University, which created the “Coronavirus Tracker®” platform, unique evidence of the power of AI collecting and analyzing large amounts of data to track the pandemic progression worldwide (7).

**Figure 1:**
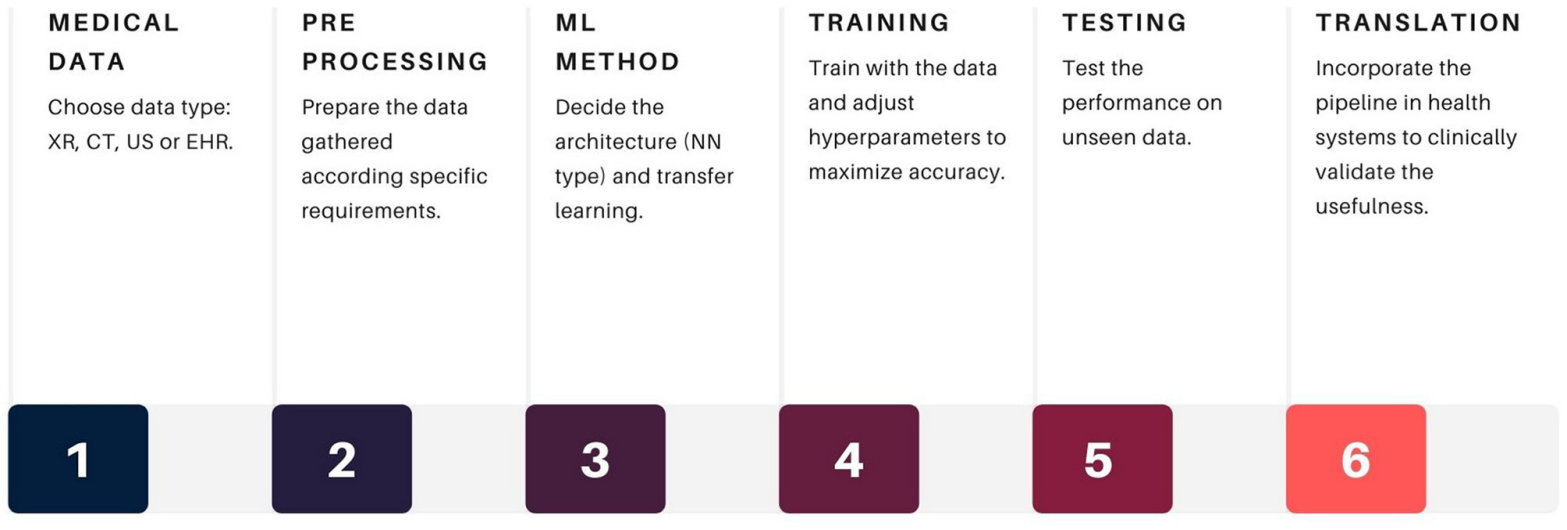
General data pipeline for COVID-19 AI applications. Source: own ellaboration. References XR, x-rays; CT, computed tomography; US, ultrasound; EHR, electroninc health records.

The data pipelines employed in AI in healthcare include steps of data gathering and processing, application of machine learning (ML) methods, and performance validation, with further translation into clinical applications with medical feedback, for example, in medical imaging. These pipelines could assist a large number of diagnostic tests and procedures performed by humans, which impact resource allocation, timing, and outcome prediction (Figure 1).

Besides accelerating and improving processes, the application of AI methods could improve outcomes and reduce costs healthcare value chain. This had never been so relevant as in the COVID-19 pandemic.

This piece aims to reflect on the value of AI during the COVID-19 pandemic, using the case of developments in medical imaging and electronic health data management.

## Application of AI in Medical Imaging

The COVID-19 pulmonary involvement characterizes by alveolar oedema with inflammatory component; pneumocyte hyperplasia and interstitial thickening can also occur as part of the reparative process. In most severe cases, different stages of consolidation are described, while the most severe include alveolar congestion, hemorrhage, necrosis, and finally lung fibrosis (8).

Lung imaging (LI) became a crucial tool to diagnose COVID-19 due to its characteristic diagnostic patterns. Three types of imaging methods are routinely used.

### Chest X-Rays (XR)

XR based methods usually identify ground-glass opacities (GGO) and pleural consolidations in COVID-19 patients with lung compromise (9). The usual AI method employs pre-trained deep neural networks [transfer learning approach in which the neural network is trained with massive general datasets first, like CIFAR 10 (10)] to classify clinical images in four patterns: normal XR, potential COVID-19, and viral or bacterial pneumonia. The overall accuracy shown in different reports was >86%, with an area under the ROC curve (AUC) >90%. Each pattern could denote higher AUC, depending on the quality of the images and the labeling (11–14).

### Chest Computed Tomography (CT)

CTs can produce more precise images, as a result, detect GGO and pulmonary involvement earlier in COVID-19. However, CT involves higher doses of radiation and requires patient handling, which could be complex (15, 16). CT automatic analysis employs transfer learning, similarly to XR, the images are classified into three patterns (excluding viral pneumonia) (14, 17–19). Additionally, with lung segmentation techniques, it is possible to separate affected regions from healthy ones and hence provide a quantification of pulmonary compromise (18). Compared to traditional XR, AI assisted CT showed better accuracy and AUC for COVID-19 case detection.

### Lung Ultrasound (LUS)

Point of Care Ultrasound drew clinicians' attention over the last 15 years, mainly because it's a portable method, could be performed at the bedside, and it is widely available in healthcare facilities. Initially, the emergency and critical care settings employed LUS to rule out pneumothorax as an alternative to standard XR, subsequently gaining field in other medical specialties (pulmonary and internal medicine) for the diagnosis, management, and monitoring of pulmonary disease (20, 21).

The normal LUS shows the A-lines, which are horizontal black waves reflecting the plane of the visceral pleura, with reverberations due to the normal presence of air (22). When lung consolidation or edema exists, the B lines (vertical, comet-tail sonographic artifacts) are noted, depending on the degree of pulmonary compromise (22, 23). LUS can be employed in COVID-19 patients to detect pulmonary compromise, including at the early stages. As LUS is not a radiation-based technique, it eliminates or reduces further exposure to X-Rays, and limits the number of staff contacts, decreasing the risk of occupational exposure to SARS-CoV-2 (24).

To distinguish a normal LUS from COVID-19 pneumonia and other causes of pneumonia (bacterial, other viruses). Automatization methods with transfer learning similar to CT and XR, are employed for the analysis of the LUS (25, 26).

## Automatic Analysis of Electronic Health Records (EHR) Using AI

Natural language processing (NLP) techniques to automatically analyze EHR allow the extraction of multiple parameters of interest as well as their tendencies, supported by the use of neural networks for their classification. Thanks to the implementation of strong disambiguation techniques to the EHR, classification accuracy improved in electronic records analysis (9). This technique is useful to assign the same meaning to the same clinical patient description that could be differentially annotated by different doctors.

Two groups of researchers analyzed more than 10000 EHRs, establishing common patterns and data models that support the prediction of severity in COVID-19 patients (27, 28). Using these published frameworks, EHRs could be analyzed and automatically extract, in a single run, parameters of interest, such as median age, gender distribution, common symptoms by age groups, disease severity, percentage of patients requiring ICU admission, among others.

## The Value Perspective of AI in the COVID-19 Pandemic

Value-based healthcare (VBH) refers to maximizing patient outcomes over the cost of delivering care (29). As value depends on the “outcome” in the entire pathway of care, they measure by the point of care and not by the volume; however, the whole healthcare system is indirectly related to those outcomes measurement (30).

The use of Value assessment frameworks (VAF) was adopted to support physicians, payers, and patients to understand the value of diverse health technologies, assessing their outcomes from the clinical, economic, and societal perspectives (31). The Core elements of value include quality-adjusted life-years gain and net costs, concepts measured traditionally from the payer or healthcare perspective. Novel elements of value include a broader societal perspective (32).

As an example, ISPOR's (The professional society for Health Economics and Outcomes Research) created a VAF which considers three novel elements of value: fear of contagion, reduction in uncertainty, and value of hope, which capture much of the attention when speaking about innovation in COVID-19, especially to assess the potential value and the appropriateness and pricing of COVID-19 treatments (31–33).

### Healthcare Systems and Payer Value

VBH considers the diagnostic pathway an intermediate outcome, and the role of medical imaging in the value chain is not fully understood (34). In contrast, the examples of the use of AI in LUS and CT processing and EHR analysis, represent outcomes-based approaches to patient care. Thereby, it should be taken into account when examining VBH in medical imaging, and outcomes-based approaches.

The advantages of AI in LUS and CT include the improvement in the time for image processing while indirectly reduce the possible exposure to SARS-CoV-2 by staff (9). In terms of patient management, by detecting pulmonary involvement at the early stages, it allows to establish intensive follow-up, monitor for signs of deterioration, and optimize patient care. Hence, it could reduce the length of stay (LOS), and avoid or limit ICU admissions, limiting the cost of the episode of care.

Data analysis from retrospective COVID-19 patients EHR may identify those at high risk of deterioration or complications: AI-driven algorithms could analyze patterns of severity, including ICU admission, mechanical ventilation requirement, LOS, and resource use, to create prediction scores applicable to patient care.

Currently, the healthcare facilities are operative in levels as before the COVID-19 surge and have to deal with COVID-19 and non-COVID-19 patients. Anticipating the resource use and needs to cope with the second wave improves cost-efficiency, which is pivotal for the financial sustainability of these healthcare institutions.

### Other Elements of Value

Understanding the geographical dynamics of COVID-19 is essential to improve the response of the healthcare system and avoid the overload of health facilities. South Korea implemented a low-cost solution for contact tracing, supported by mobile technology and data analytics. They captured data from cell phones, closed-circuit cameras, and bank transactions to track the movements of infected people and sending around text messages to their close contacts (35). Oliver et al. applied machine learning techniques and linear regression models to estimate the COVID-19 prevalence during a large-scale survey carried out in Spain during the March-April lockdown. The authors describe that results were similar to the antibody test study performed later by the government (36).

The examples above demonstrated that AI predictive analytics applied to massive testing, contact tracing, and isolation of close contacts could *reduce the uncertainty* about being infected and the *fear of contagion*, correlating with two novel elements of value (37).

Multiple drugs against COVID-19, including vaccines, are in development, opening an area of hope for COVID-19 control. *De-novo* therapies target the inhibition of the SARS-CoV-2 main protease, the spike protein receptor-binding domain, or reduce the viral replication. Repurposing drugs, an alternative path, includes testing the same targets *in-vivo* and *in-vitro* in already approved drugs (e.g., antiretrovirals), with a posterior analysis in the most suitable candidates, adjusting to additional criteria, such as binding affinity (on and off-target), toxicity levels, and elevated feasibility for synthesis (9, 38).

AI's role supports the high-speed analysis of large multidimensional data, finding the most suitable therapy candidates. It could reduce overall RD time and costs, impacting probably, in the time-to-market authorization, worth adding the value of hope for new therapies development, considering the current situation.

## Discussion

AI has shown to be a powerful tool supporting the analysis of big data from around the world during the COVID-19 pandemic. This data is relevant to identify epidemiological trends and to generate plans for both disease control and monitor the surge in healthcare facilities.

Concerning the use of AI in medical imaging, it has helped to increase the confidence levels and sensibility of X-rays and ultrasound. Not only for being able to provide automatization for the image analysis, comparable with trained medical staff, it also drastically reduced the processing times and human error. These directly impact healthcare outcomes, reducing the costs of care by increasing the number of procedures analyzed in a day, and avoiding reanalysis of some images. At this point, it's important to mention the problems of algorithms for automatic medical image analysis concerning generalization: an algorithm can have an outstanding performance on data from one hospital and then perform poorly with the same data coming from another hospital. For example, lung images might not be equal in India, where a big part of the population has tuberculosis, compared to the US that reports fewer cases (39).

Improving the classification accuracy by retraining these algorithms could be necessary due to observed demographical or clinical variations in these populations (40).

The automatic analysis of the EHR became a useful tool for screening of a high number of clinical variables at the same time, analyzing entire patient populations in hours, depending on the server capabilities. Notwithstanding, identifying the proper anonymization or de-identification pipeline is crucial to be able to operate safely with such sensible and private information contained in the EHR. Besides, during the disambiguation process for certain variables, a bottleneck occurs when identifying clinical relevance. The balance of these variables affects the global accuracy of this process (41).

To conclude, in terms of value, the main contribution of AI to the COVID-19 pandemic is the employment of automatic models that can perform patient screening, triage, diagnostic, and risk evaluation with considerable speed and efficacy and do not require a healthcare provider hands-on. Besides, integrating AI tools with big data analytics could support the assessment of COVID-19 patient care and propose further improvement in care pathways.

However, to protect private information and to ensure the appropriate clinical validation of each new data pipeline, more analysis of these methods is still required.

## Author Contributions

MC: conceptualization of the article. MC and REG: draft of the manuscript and approved the final draft. All authors contributed equally in the writing of the article.

## Conflict of Interest

REG is employed by HE-Xperts Consulting LLC. The remaining author declares that the research was conducted in the absence of any commercial or financial relationships that could be construed as a potential conflict of interest.
